# The mPower study, Parkinson disease mobile data collected using ResearchKit

**DOI:** 10.1038/sdata.2016.11

**Published:** 2016-03-03

**Authors:** Brian M. Bot, Christine Suver, Elias Chaibub Neto, Michael Kellen, Arno Klein, Christopher Bare, Megan Doerr, Abhishek Pratap, John Wilbanks, E. Ray Dorsey, Stephen H. Friend, Andrew D. Trister

**Affiliations:** 1 Sage Bionetworks, Seattle, Washington 98109, USA; 2 Center for Human Experimental Therapeutics, University of Rochester Medical Center, Rochester, New York 14642, USA

**Keywords:** Research data, Neurology, Parkinson's disease, Medical research

## Abstract

Current measures of health and disease are often insensitive, episodic, and subjective. Further, these measures generally are not designed to provide meaningful feedback to individuals. The impact of high-resolution activity data collected from mobile phones is only beginning to be explored. Here we present data from mPower, a clinical observational study about Parkinson disease conducted purely through an iPhone app interface. The study interrogated aspects of this movement disorder through surveys and frequent sensor-based recordings from participants with and without Parkinson disease. Benefitting from large enrollment and repeated measurements on many individuals, these data may help establish baseline variability of real-world activity measurement collected via mobile phones, and ultimately may lead to quantification of the ebbs-and-flows of Parkinson symptoms. App source code for these data collection modules are available through an open source license for use in studies of other conditions. We hope that releasing data contributed by engaged research participants will seed a new community of analysts working collaboratively on understanding mobile health data to advance human health.

## Background & Summary

Parkinson disease (PD) is a movement disorder related to loss of dopamine producing cells in the midbrain. Manifestations of the disease can include tremor, changes in gait, slowness (bradykinesia) and rigidity. There is significant variability in the presentation and progression of these symptoms, and while there is no known cure for PD, treatments can mitigate the effects of these symptoms. Given that typical PD patients have visits with a physician every 4–6 months, the day-to-day variability of symptoms and the effects of medications on these symptoms could reveal opportunities for interventions that might improve quality of life for those with PD. We hypothesize that more frequent quantitative assessments could lead to a better understanding of the disease heterogeneity, as well as provide individual benefit to those living with the condition. Mobile phones and other networked devices offer a unique opportunity to engage research participants without requiring physical interactions. This approach allows classic implements, such as surveys, to be administered remotely. More interestingly, sensors such as accelerometers, gyroscopes, and microphones can provide quantitative surrogates of PD symptoms with minimal or no interruption in the participant’s daily life^1^.

In March 2015, we launched mPower (https://github.com/Sage-Bionetworks/mPower), an observational smartphone-based study developed using Apple’s ResearchKit library (http://researchkit.org/) to evaluate the feasibility of remotely collecting frequent information about the daily changes in symptom severity and their sensitivity to medication in PD. These data provide the ability to explore classification of control participants and those who self-report having PD, as well as to begin to measure the severity of PD for those with the disease. There are myriad additional questions from each of the varying streams of data that will require a community of researchers to explore fully.

The study utilized a novel remote approach to enrollment in which participants self-guide through visually engaging yet complete informed consent process prior to deciding to join the study. A critical aspect of this transparent consent process is providing participants with an explicit decision point specifying if the data they donate to the study can also be used for secondary research purposes. Data described and made available here are derived from the first six months of the mPower study exclusively from those participants who chose to make their data broadly available for secondary research. We are hopeful that the data donated by mPower participants will encourage the formation of a broad, diverse, and collaborative community of PD researchers. We invite you to join this community to accelerate the research on how mobile technologies can impact PD, and together improve the quality of life for people living with PD.

## Methods

### Participant onboarding

The mPower app was made available starting in March 2015 through the Apple App Store (https://itunes.apple.com/us/app/parkinson-mpower-study-app/id972191200?mt=8) only in the United States and for iPhone 4S or newer requiring a minimum of iOS 8. Enrollment was open to individuals diagnosed with PD as well as anyone interested in participating as a control. Following download, prospective participants self-navigated through eligibility criteria (18 years of age or older, live in the United States, comfortable reading and writing on iPhone in English) and then through an interactive e-consent process (http://sagebase.org/pcc/). Ethical oversight of the study was obtained from Western Institutional Review Board. Prior to signing an electronically rendered traditional informed consent form, prospective participants had to pass a five-question quiz evaluating their understanding of the study aims, participant rights, and data sharing options. After completing the e-consent process and electronically signing the informed consent form, participants were asked for an email address to which their signed consent form was sent and allowing for verification of their enrollment in the study.

**Figure 1:**
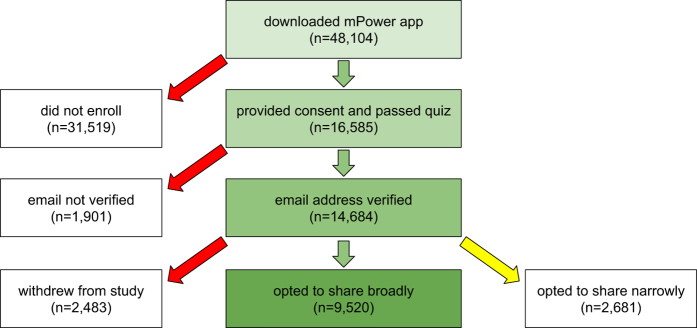
mPower study cohort description.

Participants were given the option to share their data only with the mPower study team and partners (‘share narrowly’) or to share their data more broadly with qualified researchers worldwide, and had to make an active choice to complete the consent process (no default choice was presented). The data presented here consist of all individuals who chose to have their data shared broadly (Figure 1).

### Study tasks

Once enrolled, participants were presented with a ‘dashboard’ of study tasks. Table 1 lists these tasks and their periodicity. Participants could skip any task or question within a survey at any time. A one-time baseline survey (Table 2, Data Citation 1) was the first task the participants were asked to complete. Additional standard surveys used for PD assessment, Parkinson Disease Questionnaire 8 (PDQ-8, Table 3, Data Citation 2) (ref. 2) and a subset of questions from the Movement Disorder Society Universal Parkinson Disease Rating Scale (MDS-UPDRS, Table 4, Data Citation 3) (ref. 3), were presented at baseline as well as monthly throughout the course of the study. Due to the length of the MDS-UPDRS instrument, we included only a subset of questions taken from the patient questionnaire focusing largely on self-evaluation of the motor symptoms of PD. The data obtained from these surveys is subject to the copyright holder’s license. All surveys represent self-reported outcomes and thus occasionally contain typographic errors and possibly inconsistent information.

Participants were presented four separate activities (referred to as ‘memory’, ‘tapping’, ‘voice’, and ‘walking’) which they could complete three times a day. Participants who self-identified as having a professional diagnosis of PD were asked to do these four tasks (1) immediately before taking their medication, (2) after taking their medication (when they are feeling at their best), and (3) at some other time. Participants who self-identified as not having a professional diagnosis of PD (the controls) could complete these tasks at any time during the day, with the app suggesting to complete each activity three times per day.

The memory activity (Data Citation 4) was developed to evaluate short-term spatial memory (Personal Communication with Katherine Possin). Participants observed a grid of flowers that illuminated one at and time and were then asked to replicate the illumination pattern by touching the flowers in the same order. The first release of the app (version 1.0, build 7) did not include this memory activity, however subsequent releases (starting April 21) included the activity. Included in these data are samples of where and when participants tapped on the iPhone screen and if that was a correct location as per the random pattern presented to them.

The tapping activity (Data Citation 5) measured dexterity and speed. Participants were instructed to lay their phone on a flat surface and to use two fingers on the same hand to alternatively tap two stationary points on the screen for 20 s. Included in these data are samples of where and when participants tapped on the iPhone screen as well as accelerometer readouts of how the phone was moving during the activity.

The voice activity (Data Citation 6) recorded participants’ sustained phonation by instructing them to say ‘Aaaaah’ into the microphone at a steady volume for up to 10 s. The data from this activity include audio files containing measurements from the iPhone microphone for both the 10 s of phonation as well as for the 5 s countdown leading up to the activity.

The walking activity (Data Citation 7) evaluated participants’ gait and balance. The first release of the app (version 1.0, build 7) instructed participants to walk 20 steps in a straight line, turn around, stand still for 30 s, then walk 20 steps back. Subsequent releases omitted the return walk. For each leg of this activity, data included measurements from the phone’s accelerometer and gyroscope in both raw and processed formats.

### Data collection and Distribution

The app recorded all data collected for this study through interactions with the Bridge Server, a set of web services developed and operated by Sage Bionetworks. Bridge exposes a REST style web services API designed to allow collection and management of mobile health data from a variety of apps. This service has been used by Sage and other research organizations to support a variety of health studies, including all five of the initial Research Kit apps launched in March 2015. Bridge provides apps the ability to securely create accounts for participants, and record consent and other personal information separately from study data intended to be shared with research teams.

Coded study data, consisting of survey responses and mobile sensor measurements, is exported to Synapse for distribution to researchers. Synapse^4^ is a general-purpose data and analysis sharing service where members can work collaboratively, analyze data, share insights and have attributions and provenance of those insights to share with others. Synapse is developed and operated by Sage Bionetworks as a service to the health research community. In addition to mobile health data, we have used this system to develop communities centered around shared clinical, genomic, imaging, and other types of biomedical data^5–7^. In the context of this study, Synapse also hosts the analysis of a small subset of participants that begins to show the usefulness of collecting data from PD patients in this way as well as the power of building personalized metrics of PD derived from the activities in mPower^8^.

### Code availability

The mPower mobile app (https://github.com/Sage-Bionetworks/mPower) was built using Apple’s ResearchKit framework (http://researchkit.org/), which is open source and available on GitHub (https://github.com/researchkit/researchkit). AppCore (https://github.com/ResearchKit/AppCore) is a layer built on top of ResearchKit share among the five initial ResearchKit apps. The Bridge iOS SDK (https://github.com/Sage-Bionetworks/Bridge-iOS-SDK) provides integration with Sage Bionetworks’ Bridge Server, a back-end data service designed for collection of participant donated study data (https://sagebionetworks.jira.com/wiki/display/BRIDGE/Bridge+REST+API). Example code for accessing data through Synapse as well as code used for summary statistics and generating figures for this paper are also made available (https://github.com/Sage-Bionetworks/mPower-sdata).

## Data Records

**Figure 2:**
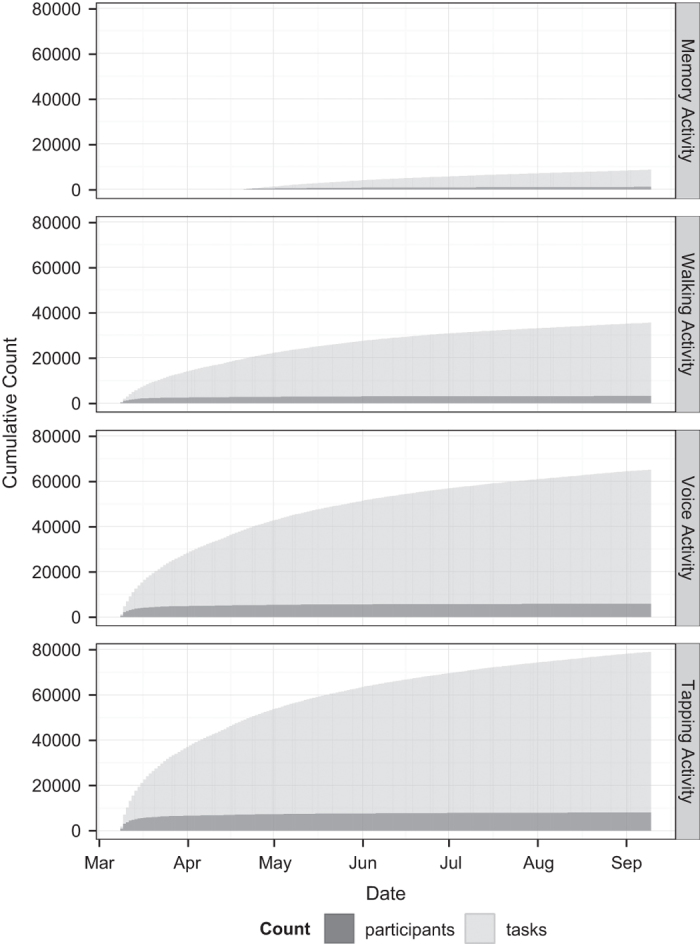
Cumulative participation for activities over time.

**Figure 3:**
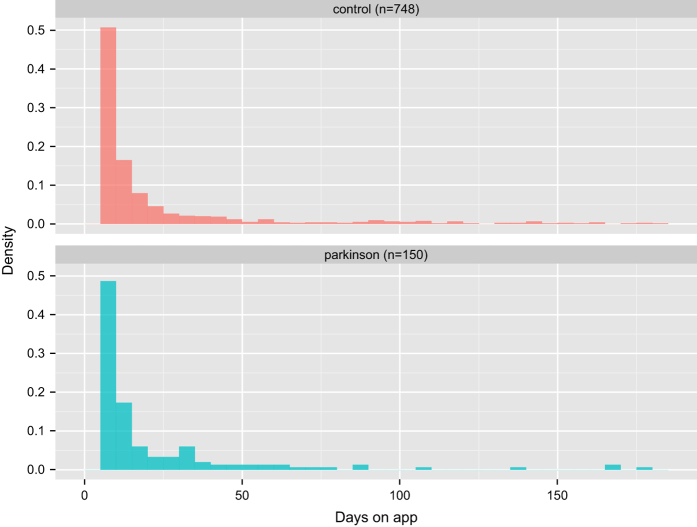
Participation shown as number of days visiting the app for all participants who completed at least one task on five separate days.

A total of 9,520 participants consented to the study and agreed to share their data broadly with the research community. 8,320 participants completed at least one survey or task after joining the study. Of the 6,805 participants who completed the enrollment survey, 1,087 self-identified as having a professional diagnosis of PD while 5,581 did not (137 opted not to answer the question). Data contributed by participants for each survey and activity are enumerated in Table 1 and cumulative tasks completed for each activity are presented in Figure 2. Due to the nature of the study, follow up is nonuniform across individuals, however 898 participants contributed data at least five separate days over the course of the first six months of the study. The number of days participants contributed data were similarly distributed between those who self-reported as having a diagnosis of PD and those participating as controls (Figure 3).

All coded data sets (Table 1, Data Citation 1, Data Citation 2, Data Citation 3, Data Citation 4, Data Citation 5, Data Citation 6, Data Citation 7) are stored and accessible via the Synapse platform in a public project with associated metadata and documentation (https://www.synapse.org/mPower).

## Technical Validation

The data provided herein are derived from Apple iPhone devices with proprietary technical validation. We do not provide test-retest nor other technical validation data sets here, however others have reported technical validation of the coreMotion sensor in a different context^9^.

## Usage Notes

Due to the novel nature and collection method for these data, governance structures have been put in place in order to respect the balance between the desire of participants to share their data with qualified researchers and the respect for privacy of those participants.

Researchers who are interested in accessing these data need to complete the following steps:

1. Have a Synapse account (https://synapse.org)
2. Have their Synapse User Profile validated by the Synapse Access and Compliance Team (ACT)
3. Become a Synapse Certified user by completing a short quiz (https://www.synapse.org/#!Quiz:Certification)
4. Submit an Intended Data Use statement
5. Agree to the Conditions for Use associated with each data source (see DOIs for each data source)

While certain data types may have additional Conditions for Use (e.g. survey copyrights), the overarching Conditions for Use are as follows:

- You confirm that you will not attempt to re-identify research participants for any reason, including for re-identification theory research.
- You reaffirm your commitment to the Synapse Awareness and Ethics Pledge.
- You agree to abide by the guiding principles for responsible research use and data handling as described in the Synapse Governance documents (https://www.synapse.org/#!Help:Governance).
- You commit to keeping these data confidential and secure.
- You agree to use these data exclusively as described in your submitted Intended Data Use statement.
- You understand that these data may not be used for commercial advertisement or to re-contact research participants.
- You agree to report any misuse or data release, intentional or inadvertent to the ACT within 5 business days by emailing act@sagebase.org.
- You agree to publish findings in open access publications.
- You promise to acknowledge the research participants as data contributors and mPower study investigators on all publication or presentation resulting from using these data as follows: ‘These data were contributed by users of the Parkinson mPower mobile application as part of the mPower study developed by Sage Bionetworks and described in Synapse [https://www.synapse.org/mPower].’

Examples of client interactions with these data are provided in GitHub: https://github.com/Sage-Bionetworks/mPower-sdata

## Additional Information

**How to cite**: Bot, B. M. *et al.* The mPower Study, Parkinson Disease Mobile Data Collected Using ResearchKit. *Sci. Data* 3:160011 doi: 10.1038/sdata.2016.11 (2016).

## Supplementary Material



## Figures and Tables

**Table 1:** Data available for each survey and activity completed by study participants.

**Task name**	**Type of task and schedule**	**Citation**	**unique participants**	**unique tasks**
Demographics	Survey—once	Data Citation 1	6,805	6,805
PDQ8	Survey—monthly	Data Citation 2	1,334	1,641
UPDRS	Survey—monthly	Data Citation 3	2,024	2,305
Memory	Activity—t.i.d.	Data Citation 4	968	8,569
Tapping	Activity—t.i.d.	Data Citation 5	8,003	78,887
Voice	Activity—t.i.d.	Data Citation 6	5,826	65,022
Walking	Activity—t.i.d.	Data Citation 7	3,101	35,410

**Table 2:** Demographics Survey.

**Question**	**Variable name**	**Variable details**
How old are you?	age	integer
What is your sex?	gender	one of {‘Female’, ‘Male’, ‘Prefer not to answer’}
Which race do you identify with?	race	check all that apply {‘Black or African’, ‘Latino/Hispanic’, ‘Native American’, ‘Pacific Islander’, ‘Middle Eastern’, ‘Caribbean’, ‘South Asian’, ‘East Asian’, ‘White or Caucasian’, ‘Mixed’}
What is the highest level of education that you have completed?	education	one of {‘2-year college degree’, ‘4-year college degree’, ‘Doctoral Degree’, ‘High School Diploma/GED’, ‘Master's Degree’, ‘Some college’, ‘Some graduate school’, ‘Some high school’}
What is your current employment status?	employment	one of {‘A homemaker’, ‘A student’, ‘Employment for wages’, ‘Out of work’, ‘Retired’, ‘Self-employed’, ‘Unable to work’}
What is your current marital status?	maritalStatus	one of {‘Divorced’, ‘Married or domestic partnership’, ‘Other’, ‘Separated’, ‘Single, never married’, ‘Widowed’}
Are you a spouse, partner or care-partner of someone who has Parkinson disease?	are-caretaker	one of {‘true’, ‘false’}
Have you ever participated in a research study or clinical trial on Parkinson disease before?	past-participation	one of {‘true’, ‘false’}
How easy is it for you to use your smartphone?	smartphone	one of {‘Difficult’, ‘Easy’, ‘Neither easy nor difficult’, ‘Very Difficult’, ‘Very easy’}
Do you ever use your smartphone to look for health or medical information online?	phone-usage	one of {‘true’, ‘false’, ‘Not sure’}
Do you use the Internet or email at home?	home-usage	one of {‘true’, ‘false’}
Do you ever use the Internet to look for health or medical information online?	medical-usage	one of {‘true’, ‘false’}
Did you happen to do this yesterday, or not?	medical-usage-yesterday	one of {‘true’, ‘false’, ‘don’t know’}
Do you ever use your smartphone to participate in a video call or video chat?	video-usage	one of {‘true’, ‘false’}
Have you been diagnosed by a medical professional with Parkinson disease?	professional-diagnosis	one of {‘true’, ‘false’}
In what year did your movement symptoms begin?	onset-year	integer input
In what year were you diagnosed with Parkinson disease?	diagnosis-year	integer input
In what year did you begin taking Parkinson disease medication? Type in 0 if you have not started to take Parkinson medication.	medication-start-year	integer input
What kind of health care provider currently cares for your Parkinson disease?	healthcare-provider	one of {‘Don't know’, ‘General Neurologist (non-Parkinson Disease specialist)’, ‘Nurse Practitioner or Physician's Assistant’, ‘Other’, ‘Parkinson Disease/Movement Disorder Specialist’, ‘Primary Care Doctor’}
Have you ever had Deep Brain Stimulation?	deep-brain-stimulation	one of {‘true’, ‘false’}
Have you ever had any surgery for Parkinson disease, other than DBS?	surgery	one of {‘true’, ‘false’}
Have you ever smoked?	smoked	one of {‘true’, ‘false’}
How many years have you smoked?	years-smoking	integer input
On average, how many packs did you smoke each day?	packs-per-day	one of {1, 2, 3, 4, 5}
When is the last time you smoked (put today’s date if you are still smoking)?	last-smoked	year last smoked
Has a doctor ever told you that you have any of the following conditions? Please check all that apply.	health-history	Multiple choice from {‘Acute Myocardial Infarction/Heart Attack’, ‘Alzheimer Disease or Alzheimer dementia’, ‘Atrial Fibrillation’, ‘Anxiety’, ‘Cataract’, ‘Kidney Disease’, ‘Chronic Obstructive Pulmonary Disease (COPD) or Asthma’, ‘Heart Failure/Congestive Heart Failure’, ‘Diabetes or Prediabetes or High Blood Sugar’, ‘Glaucoma’, ‘Hip/Pelvic Fracture’, ‘Ischemic Heart Disease’, ‘Depression’, ‘Osteoporosis’, ‘Rheumatoid Arthritis’, ‘Dementia’, ‘Stroke/Transient Ischemic Attack (TIA)’, ‘Breast Cancer’, ‘Colorectal Cancer’, ‘Prostate Cancer’, ‘Lung Cancer’, ‘Endometrial/Uterine Cancer’, ‘Any other kind of cancer OR tumor’,‘Head Injury with Loss of Consciousness/Concussion’, ‘Urinary Tract infections’, ‘Obstructive Sleep Apnea’, ‘Schizophrenia or Bipolar Disorder’, ‘Peripheral Vascular Disease’, ‘High Blood Pressure/Hypertension’, ‘Fainting/Syncope’, ‘Alcoholism’, ‘Multiple Sclerosis’, ‘Impulse control disorder’, ‘AIDS or HIV’, ‘Liver Disease’, ‘Leukemia or Lymphoma’, ‘Ulcer Disease’, ‘Connective Tissue Disease’, ‘Coronary Artery Disease’, ‘Anemia’,‘Asthma’}

**Table 3:** PDQ8 Survey.

**Question**	**Variable name**	**Variable details**
Due to Parkinson’s disease, how often during the last month have you had difficulty getting around in public?	PDQ8-1	one of: {‘Never’, ‘Occasionally’, ‘Sometimes’, ‘Often’, ‘Always’}
Due to Parkinson’s disease, how often during the last month have you had difficulty dressing yourself?	PDQ8-2	one of: {‘Never’, ‘Occasionally’, ‘Sometimes’, ‘Often’, ‘Always’}
Due to Parkinson’s disease, how often during the last month have you felt depressed?	PDQ8-3	one of: {‘Never’, ‘Occasionally’, ‘Sometimes’, ‘Often’, ‘Always’}
Due to Parkinson’s disease, how often during the last month have you had problems with your close personal relationships?	PDQ8-4	one of: {‘Never’, ‘Occasionally’, ‘Sometimes’, ‘Often’, ‘Always’}
Due to Parkinson’s disease, how often during the last month have you had problems with your concentration, e.g. when reading or watching TV?	PDQ8-5	one of: {‘Never’, ‘Occasionally’, ‘Sometimes’, ‘Often’, ‘Always’}
Due to Parkinson’s disease, how often during the last month have you felt unable to communicate with people properly?	PDQ8-6	one of: {‘Never’, ‘Occasionally’, ‘Sometimes’, ‘Often’, ‘Always’}
Due to Parkinson’s disease, how often during the last month have you had painful muscle cramps or spasms?	PDQ8-7	one of: {‘Never’, ‘Occasionally’, ‘Sometimes’, ‘Often’, ‘Always’}
Due to Parkinson’s disease, how often during the last month have you felt embarrassed in public due to having Parkinson’s disease?	PDQ8-8	one of: {‘Never’, ‘Occasionally’, ‘Sometimes’, ‘Often’, ‘Always’}

**Table 4:** UPDRS Survey.

**Question**	**Variable name**	**Variable details**
How good or bad is your health TODAY (0 means the worst health you can imagine, 100 means the best health you can imagine)?	EQ-5D1	sliding scale from 0-100
Over the past week, how many times did you do the following kinds of exercise for more than 15 minutes? Strenuous exercise (heart beats rapidly):	GELTQ-1a	integer input
Over the past week, how many times did you do the following kinds of exercise for more than 15 minutes? Moderate exercise (not exhausting):	GELTQ-1b	integer input
Over the past week, how many times did you do the following kinds of exercise for more than 15 minutes? Minimal effort:	GELTQ-1c	integer input
During your leisure time in the past week, how often do you engage in any regular activity long enough to work up a sweat (heart beats rapidly)?	GELTQ-2	one of: {‘Often’, ‘Sometimes’, ‘Never/Rarely’}
Over the past week have you had problems remembering things, following conversations, paying attention, thinking clearly, or finding your way around the house or in town?	MDS-UPDRS1.1	one of: {‘Normal’, ‘Slight’, ‘Mild’, ‘Moderate’, ‘Severe’} mapping to {0, 1, 2, 3, 4}
Over the past week have you felt low, sad, hopeless, or unable to enjoy things?	MDS-UPDRS1.3	one of: {‘Normal’, ‘Slight’, ‘Mild’, ‘Moderate’, ‘Severe’} mapping to {0, 1, 2, 3, 4}
Over the past week have you felt nervous, worried or tense?	MDS-UPDRS1.4	one of: {‘Normal’, ‘Slight’, ‘Mild’, ‘Moderate’, ‘Severe’} mapping to {0, 1, 2, 3, 4}
Over the past week, have you felt indifferent to doing activities or being with people?	MDS-UPDRS1.5	one of: {‘Normal’, ‘Slight’, ‘Mild’, ‘Moderate’, ‘Severe’} mapping to {0, 1, 2, 3, 4}
Over the past week, have you had trouble going to sleep at night or staying asleep through the night? Consider how rested you felt after waking up in the morning.	MDS-UPDRS1.7	one of: {‘Normal’, ‘Slight’, ‘Mild’, ‘Moderate’, ‘Severe’} mapping to {0, 1, 2, 3, 4}
Over the past week, have you had trouble staying awake during the daytime?	MDS-UPDRS1.8	one of: {‘Normal’, ‘Slight’, ‘Mild’, ‘Moderate’, ‘Severe’} mapping to {0, 1, 2, 3, 4}
Over the past week, have you had problems with your speech?	MDS-UPDRS2.1	one of: {‘Normal’, ‘Slight’, ‘Mild’, ‘Moderate’, ‘Severe’} mapping to {0, 1, 2, 3, 4}
Over the past week, have you usually had troubles handling your food and using eating utensils? For example, do you have trouble handling finger foods or using forks, knives, spoons, chopsticks?	MDS-UPDRS2.4	one of: {‘Normal’, ‘Slight’, ‘Mild’, ‘Moderate’, ‘Severe’} mapping to {0, 1, 2, 3, 4}
Over the past week, have you usually had problems dressing? For example, are you slow or do you need help with buttoning, using zippers, putting on or taking off your clothes or jewelry?	MDS-UPDRS2.5	one of: {‘Normal’, ‘Slight’, ‘Mild’, ‘Moderate’, ‘Severe’} mapping to {0, 1, 2, 3, 4}
Over the past week, have you usually been slow or do you need help with washing, bathing, shaving, brushing teeth, combing your hair, or with other personal hygiene?	MDS-UPDRS2.6	one of: {‘Normal’, ‘Slight’, ‘Mild’, ‘Moderate’, ‘Severe’} mapping to {0, 1, 2, 3, 4}
Over the past week, have people usually had trouble reading your handwriting?	MDS-UPDRS2.7	one of: {‘Normal’, ‘Slight’, ‘Mild’, ‘Moderate’, ‘Severe’} mapping to {0, 1, 2, 3, 4}
Over the past week, have you usually had trouble doing your hobbies or other things that you like to do?	MDS-UPDRS2.8	one of: {‘Normal’, ‘Slight’, ‘Mild’, ‘Moderate’, ‘Severe’} mapping to {0, 1, 2, 3, 4}
Over the past week, do you usually have trouble turning over in bed?	MDS-UPDRS2.9	one of: {‘Normal’, ‘Slight’, ‘Mild’, ‘Moderate’, ‘Severe’} mapping to {0, 1, 2, 3, 4}
Over the past week, have you usually had shaking or tremor?	MDS-UPDRS2.10	one of: {‘Normal’, ‘Slight’, ‘Mild’, ‘Moderate’, ‘Severe’} mapping to {0, 1, 2, 3, 4}
Over the past week, have you usually had problems with balance and walking?	MDS-UPDRS2.12	one of: {‘Normal’, ‘Slight’, ‘Mild’, ‘Moderate’, ‘Severe’} mapping to {0, 1, 2, 3, 4}
Over the past week, on your usual day when walking, do you suddenly stop or freeze as if your feet are stuck to the floor?	MDS-UPDRS2.13	one of: {‘Normal’, ‘Slight’, ‘Mild’, ‘Moderate’, ‘Severe’} mapping to {0, 1, 2, 3, 4}
